# Role of Mast Cells in Oral Lichen Planus and Oral Lichenoid Reactions

**DOI:** 10.1155/2018/7936564

**Published:** 2018-01-17

**Authors:** Suganya Ramalingam, Narasimhan Malathi, Harikrishnan Thamizhchelvan, Narasimhan Sangeetha, Sharada T Rajan

**Affiliations:** ^1^Department of Oral Pathology, Faculty of Dental Sciences, Sri Ramachandra Medical College and Research Institute (Deemed to Be University), Chennai, India; ^2^Oral and Craniofacial Health Sciences, College of Dental Medicine, University of Sharjah, Sharjah, UAE

## Abstract

**Introduction:**

Oral lichen planus (OLP) is a chronic T cell mediated disease of oral mucosa, skin, and its appendages with a prevalence of 0.5 to 2.6% worldwide. Oral lichenoid reactions (OLR) are a group of lesions with diverse aetiologies but have clinical and histological features similar to OLP, thereby posing a great challenge in differentiating both lesions. Mast cells are multifunctional immune cells that play a major role in the pathogenesis of lichen planus by release of certain chemical mediators. Increased mast cell densities with significant percentage of degranulation have been observed as a consistent finding in pathogenesis of oral lichen planus.

**Aim:**

The current study was aimed at quantifying the mast cells in histopathological sections of OLP and OLR thereby aiding a means of distinguishing these lesions.

**Materials and Methods:**

The study group involved 21 cases of oral lichen planus, 21 cases of oral lichenoid reactions, and 10 control specimens of normal buccal mucosa. All the cases were stained with Toluidine Blue and routine haematoxylin and eosin and the mast cells were quantified.

**Statistical Analysis Used:**

The results were analyzed using the Kruskal–Wallis test and an intergroup analysis was performed using Mann–Whitney *U* test.

**Conclusion:**

The number of mast cells showed an increased value in oral lichen planus when compared to oral lichenoid reaction and thus an estimation of mast cells count could aid in distinguishing OLP from OLR histopathologically.

## 1. Introduction

Lichen planus is a chronic mucocutaneous T cell mediated disease that usually involves the oral mucosa [1]. An antigen specific cell mediated immune response initiated by various chemokines and extracellular matrix proteins act through different pathways to cause degeneration of basal keratinocytes in the oral epithelium [2]. Clinically, OLP almost always occurs bilaterally. Though these lesions exhibit various clinical forms, namely, ulcerated, papular, atrophic, bullous, plaque-like, and reticular lesions, the highly characteristic feature of OLP showing fine white lines forming a lace-like network called Wickham's striae should always be present to define it as LP [3]. However, the presence of Wickham's striae alone does not usually designate the lesion as oral lichen planus, because the diagnosis of OLP is always clinicopathological [4].

The WHO criteria (1978) for histopathological diagnosis of OLP include thickened ortho- or parakeratinised epithelium, liquefaction degeneration of basal layer, well-defined juxtaepithelial lymphocytic infiltration, and Civatte bodies in basal epithelium and lamina propria [3].

Oral lichenoid reactions are clinical and histological analogues to oral lichen planus. They appear as white hyperkeratotic thickened lesions often related to drugs, dental restorative materials, graft versus host disease, and medications. These lesions occur in atypical sites that usually has straight topographic relation to the causative agent. Similar to oral lichen planus, the lichenoid reactions also occur in various forms like erythematous, reticular, plaque-like, and atrophic [5].

Histopathologically oral lichenoid reactions show more diffuse subepithelial inflammatory infiltrate which extends much below into the connective tissue and is more lymphohistiocytic compared to mixed inflammatory infiltration [5].

Mast cells are granular, bone-marrow derived, mobile leucocytes with wide range of functions including inflammation, immune modulation, tissue repair, and remodelling. On response to immunologic and nonimmunologic stimuli, they release preformed mediators, vasoactive amines, cytokines, and enzymes via granules [6]. The interaction between T cells and secretions from mast cells plays an important role in the regulation of immune responses emphasizing the immunopathogenesis of OLP.

Antigenically altered basal epithelial cells stimulate the mucosal mast cells through RANTES. Stimulation of mast cells presents the antigen to the T cells which in sequence causes activation and degranulation of mast cell and release of various factors like TNF-*α*, a series of interleukins (IL-3, IL-4, IL-5, IL-6, IL-8, IL-10, IL-11, IL-13, and IL-16), chymase, and tryptase. Both chymase and tryptase which are derivative of mast cell along with matrix metalloproteinase which is derivative of T cell lead to the breakup of basement membrane of the epithelium thereby exposing the basal keratinocytes to the cytotoxic CD8+ T cells leading to their apoptosis [7].

One of the important causes for oral lichenoid reactions could be allergic hypersensitivity reaction in which main causative phenomena occur by degranulation of mast cells [8].

The present study was designed for quantifying mast cells in oral lichenoid reactions and oral lichen planus to understand the potential function of mast cells in the pathogenesis of these diseases.

## 2. Materials and Methods

**Figure 1:**
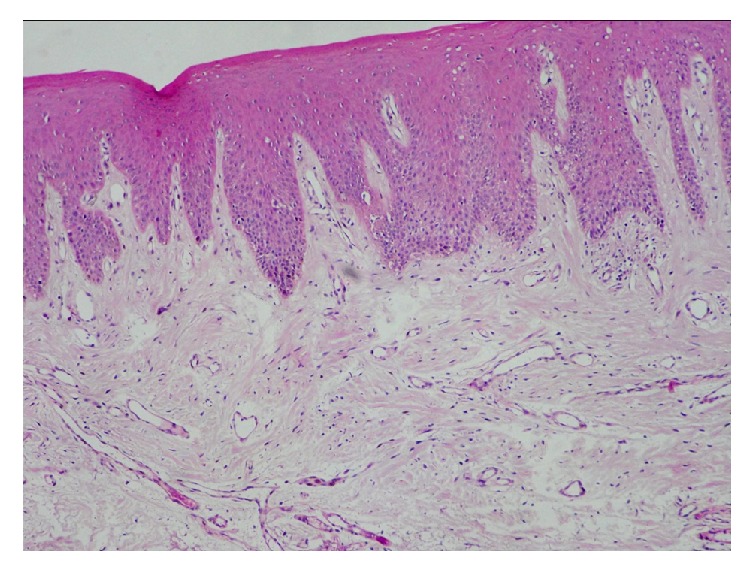
Photomicrograph of the section shows normal buccal mucosa (H&E 10x).

**Figure 2:**
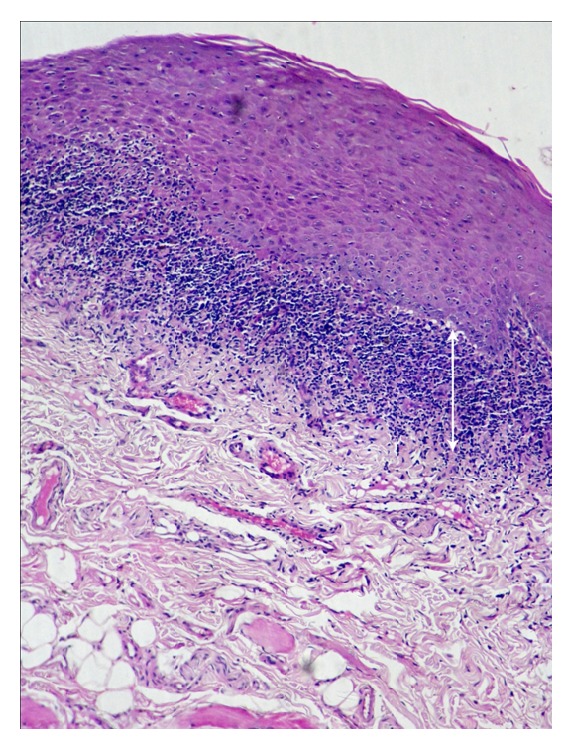
Photomicrograph of the section shows parakeratotic stratified squamous epithelium with juxtaepithelial band of inflammatory infiltration in oral lichen planus (H&E 10x). The arrows refer to juxtaepithelial band of inflammatory infiltration in oral lichen planus.

**Figure 3:**
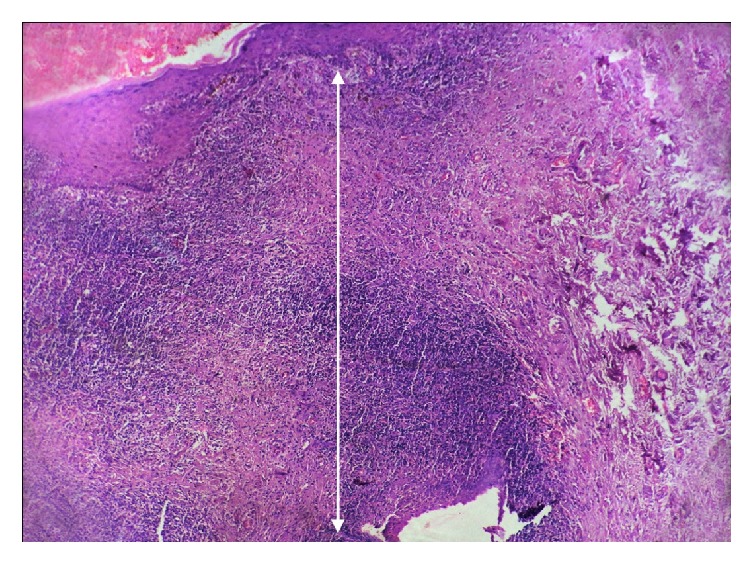
Photomicrograph of the section shows parakeratotic stratified squamous epithelium with basal cell degeneration. The arrows refer to inflammatory infiltration extending deep into reticular zone of lamina propria in oral lichenoid reaction (H&E 20x).

**Figure 4:**
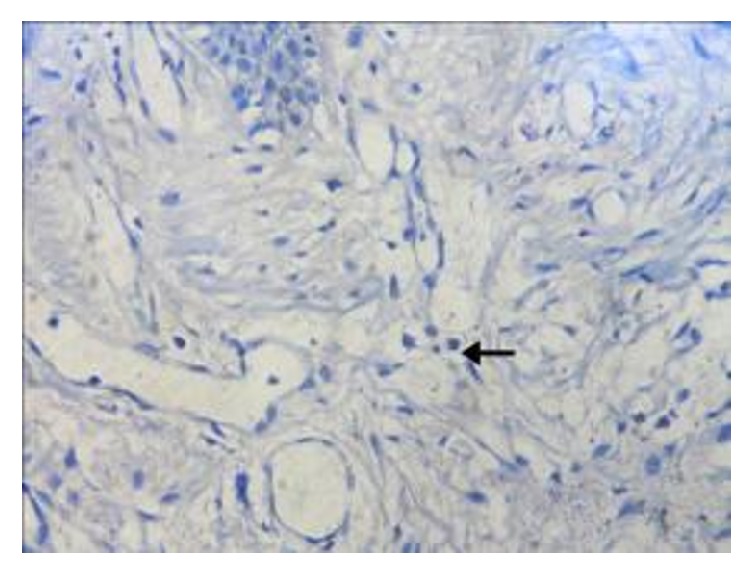
Photomicrograph of the section shows normal buccal mucosa (Toluidine Blue 10x). The arrows refer to mast cells.

**Figure 5:**
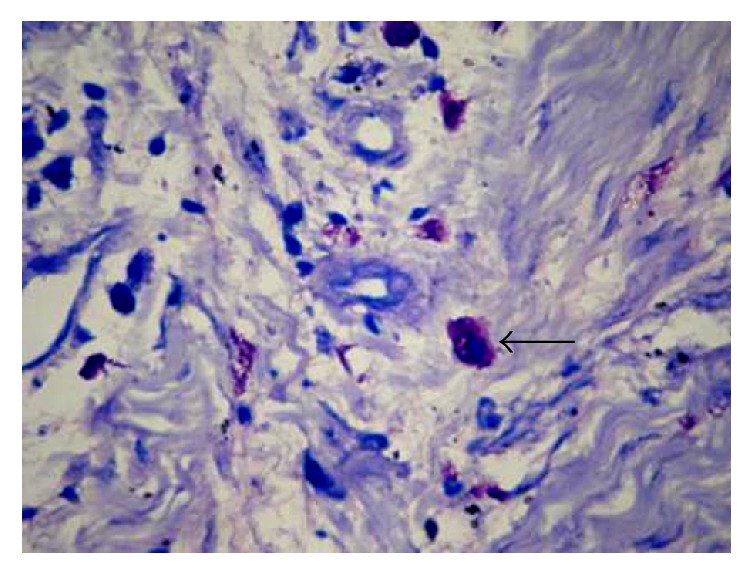
Photomicrograph of the section shows intact mast cells in oral lichen planus (Toluidine Blue 40x). The arrows refer to intact mast cells in oral lichen planus.

**Figure 6:**
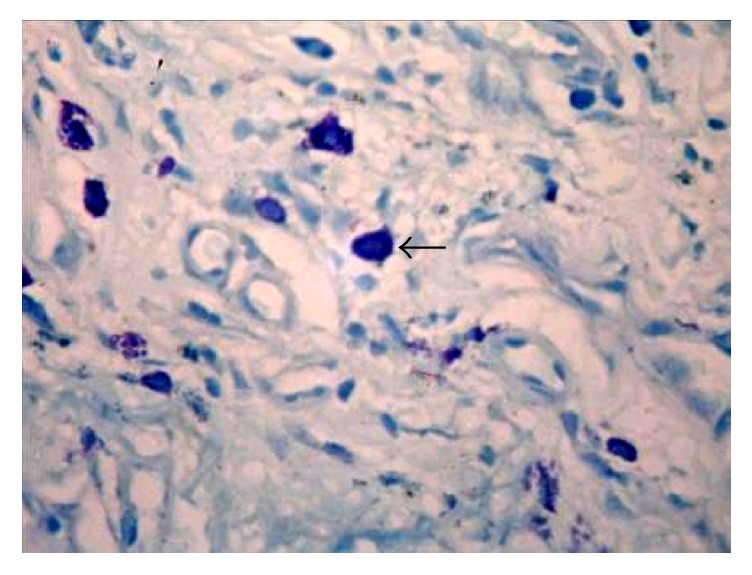
Photomicrograph of the section shows intact mast cells in oral lichenoid reaction (H&E 40x). The arrow refers to intact mast cells.

The current study involved formalin-fixed paraffin-embedded tissue sections of histopathologically diagnosed cases of oral lichen planus (*n* = 21) and oral lichenoid reactions (*n* = 21) from the archives of Department of Oral Pathology. Demographic data and history of the cases were also retrieved from Department of Medical Records. Normal buccal mucosa tissues (*n* = 10) from volunteers with no oral lesions and no associated oral habits were also obtained after taking informed consent and processed in the same way as routine pathological specimens. All study samples were stained with routine histopathological haematoxylin and eosin stain (Figures 1–3) and a special Toluidine Blue stain (Figures 4–6).

### 2.1. Special Stain: Toluidine Blue

1 gm of Toluidine Blue O (sigma) and 100 mL of 70% alcohol were used to prepare Toluidine Blue stock solution. One percent sodium chloride was freshly prepared and pH was adjusted to 2.0–2.5 using glacial acetic acid. Working solution with pH of 2.3 was prepared with 5 mL of Toluidine Blue stock solution and 45 mL of 1% sodium chloride. The working solution was prepared fresh and was discarded after use each time.

By using rotary microtome, 4 *μ*m tissue sections were cut and transferred to egg albumin coated microscopic slides. The slides were kept on the slide warmer at 58°C for 15 minutes and were deparaffinised by immersing in 3 changes of fresh xylene each for 10 minutes. Further they were dehydrated in 3 changes of absolute alcohol for 5 minutes each and washed in running tap water for 10 minutes. The sections were treated with working solution of Toluidine Blue for 2-3 minutes followed by three changes of distilled water wash. The slides were then dehydrated quickly in 95% alcohol and 2 changes of absolute alcohol and were cleared in 2 changes of xylene and mounted with DPX. The mast cells stained deep purple against a blue background. Ten higher magnifications fields (40x) were selected at random and mast cells were counted using a compound light microscope.

## 3. Observation and Results

The age group of study samples range from 18 to 70 years. The mean age of the patients in Group I, Group II, and Group II was 29.2 years, 43.2 years, and 43.8 years, respectively. The male: female ratio among Group I, Group II, and Group III was 1 : 1, 1.6 : 1, and 1 : 1.6, respectively. The study sample included 4 different sites, buccal mucosa, tongue, lip, and gingiva. All the normal tissues were from the buccal mucosa. Wickham's striae were present in 95.2% of oral lichen planus and 57.1% of oral lichenoid reactions which were statistically significant (*p* value: 0.004) (Table 1).

**Figure 7:**
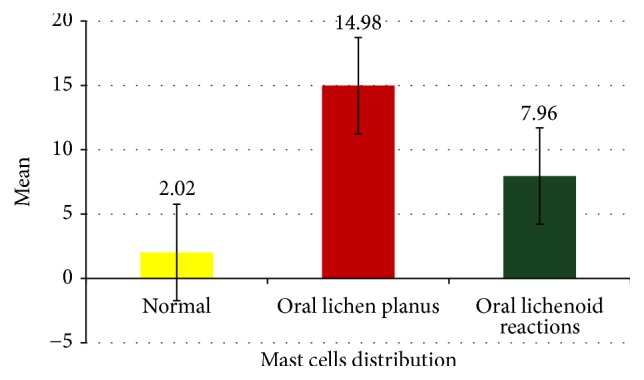
Mast cells distribution between study group.

The numbers of mast cells were significantly high in oral lichen planus (mean: 14.98) compared to oral lichenoid reactions (mean: 7.96) and normal oral mucosal tissues (mean: 2.02) (Figure 7). Comparisons of mean number of mast cells among three groups were done using Kruskal–Wallis test and it was statistically significant (*p* value: 0.001) (Table 2). Intergroup analysis was done using Mann–Whitney *U* test which was also statistically significant (*p* value: 0.0002, 0.0004, and 0.000001) (Table 3).

## 4. Discussion

Oral lichen planus is a T cell mediated autoimmune disease in which autocytotoxic CD8+ T cells activate the apoptosis of oral epithelial cells [9]. Clinically, oral lichenoid reactions may mimic OLP; nevertheless they are unilateral in distribution unlike bilateral occurrence in OLP. These lesions might be associated with a known aetiology; however they are analogous to oral lichen planus both clinically and histologically [10]. Mast cells have also been demonstrated in the lamina propria of oral lichenoid reaction but however the role of these cells in pathogenesis of OLR is not well understood like OLP. Various studies have provided substantial data to support the responsibility of mast cells in the pathogenesis of oral lichen planus and oral lichenoid reaction. Our study was aimed at quantifying mast cells as a distinguishing factor between OLP and OLR histopathologically. Toluidine Blue which has high affinity for the metachromatic granules in the mast cells was chosen to specifically recognize these cells in the study samples.

Oral lichen planus occurs mainly in adults above 40 years with female predilection [11]. However our sample showed slight increase in male predilection. This could be attributed to smaller sample size. A high female predilection is also noted in OLR irrespective of its etiology which matches in our study [12]. A wide variety of studies have concluded that the buccal mucosa is the most common site of occurrence of OLP and OLR in the oral cavity [13–15]. Among the 42 cases studied, about 73% of lesions were present on the buccal mucosa which is consistent with the above studies. A steady increase in the quantity of mast cells from normal oral tissues to OLR to OLP was noted among the samples. The mast cell count was highest in OLP cases compared to OLR suggesting a vital role demonstrated by mast cells in the pathogenesis of these lesions. Similar findings consistent with our study were noted in several other studies conducted by various authors [8, 16, 17]. In OLP, degranulation of mast cells releases proinflammatory mediators such as chymase, TNF-*α*, and tryptase. Release of TNF-*α* from degranulation of mast cells can upregulate RANTES from T cells leading to the activation and secretion of matrix metalloproteinases by T cells from lesions which are responsible for the degradation of basement membrane. At the areas of basement membrane disruption in OLP, presence of both mast cells and intraepithelial CD8^+^ T cells suggests its role in pathogenesis of OLP. This concept emphasizes how mast cells and T cells work together for the growth of lesion chronicity in OLP; thus it recognizes that breakdown of matrix may help migration of T cell from the vasculature to epithelium and lamina propria [11, 16]. In OLR, the exposure to exogenic antigens triggers a hyperimmune type of response which mobilizes mast cells to the affected site, which in turn causes keratinocytes apoptosis. Therefore, from our study, the difficulties in separation of the two conditions in the perspective of overlapping in the range of the values have been clearly explained. However, unlike OLP, the withdrawal of the causative agent leads to a reduction in the mast cells in OLR justifying a decreased number of mast cells in OLR compared to OLP [18].

## 5. Conclusion

Our study revealed that mast cell count is increased in OLP compared to OLR thereby guiding us to propose that mast cell count can be used as one of the essential histopathological elements in the differentiation of OLP and OLR.

## Figures and Tables

**Table 1:** Chi-square test.

Variable	Chi-square value	*p* value
Wickham's striae	8.4	0.004 (significant)

**Table 2:** Comparison of mast cell distribution among study groups using Kruskal–Wallis test.

Group	*N*	Mean mast cell count	Range	Standard deviation	*p* value
Group I	10	2.02	0.9–3.1	1.18	*0.001 (significant)*
Group II	21	14.98	7.9–33.4	5.16	
Group III	21	7.96	1.2–17.4	5.04	

*Note*.  *p* value is significant below the value of 0.05.

**Table 3:** Intergroup comparison of mast cell distribution using Mann–Whitney *U* test.

S. number	Comparison of groups	Median	Mann–Whitney *U* test	*p* value
(1)	*Group II versus Group III*			
	Group II	14.4	368.5	*0.0002 (significant)*
	Group III	6.6	72.5	

(2)	*Group III versus Group I*			
	Group III	6.6	188.5	*0.0004 (significant)*
	Group I	1.5	21.5	

(3)	*Group II versus Group I*			
	Group II	14.4	210	*0.000001 (significant)*
	Group I	1.5	0	

*p* value of <0.017 was considered as statistically significant.
